# The Intersection between Immune System and Idiopathic Pulmonary Fibrosis—A Concise Review

**DOI:** 10.70322/fibrosis.2025.10004

**Published:** 2025-02-18

**Authors:** Hongli Liu, Huachun Cui, Gang Liu

**Affiliations:** Division of Pulmonary, Allergy, and Critical Care Medicine, Department of Medicine, University of Alabama at Birmingham, Birmingham, AL 35294, USA

**Keywords:** IPF, Macrophage, Innate immunity, Adaptive immunity, Clinical trial

## Abstract

Idiopathic pulmonary fibrosis (IPF) is marked by progressive alveolar destruction, impaired tissue regeneration, and relentless fibrogenesis, culminating in respiratory failure and death. A diverse array of resident and non-resident cells within the lung contribute to disease pathogenesis. Notably, immune cells, both resident and recruited, respond to cues from sites of lung injury by undergoing phenotypic transitions and producing a wide range of mediators that influence, initiate, or dictate the function, or dysfunction, of key effector cells in IPF pathology, such as alveolar epithelial cells, lung fibroblasts, and capillary endothelial cells. The role of the immune system in IPF has undergone an interesting evolution, oscillating from initial enthusiasm to skepticism, and now to a renewed focus. This shift reflects both the past failures of immune-targeting therapies for IPF and the unprecedented insights into immune cell heterogeneity provided by emerging technologies. In this article, we review the historical evolution of perspectives on the immune system’s role in IPF pathogenesis and examine the lessons learned from previous therapeutic failures targeting immune responses. We discuss the major immune cell types implicated in IPF progression, highlighting their phenotypic transitions and mechanisms of action. Finally, we identify key knowledge gaps and propose future directions for research on the immune system in IPF.

## 1. Introduction

Idiopathic pulmonary fibrosis (IPF) is a progressive, fibrotic lung disease with a poor prognosis and limited treatment options, marked by aberrant wound healing and excessive extracellular matrix deposition. Despite the initial failure of immunosuppressive therapy in IPF patients, recent evidence highlights a significant role of immune dysregulation in IPF pathogenesis. Immune cells exhibit altered phenotypes in IPF, influencing disease progression through complex interactions with fibroblasts and extracellular matrix components. Immunological contributions to fibrosis are increasingly understood as multifaceted, with immune cell recruitment, activation, and polarization varying across disease stages. This review synthesizes current knowledge on the immune system’s role in IPF, with particular emphasis on macrophages/monocytes, T helper cells, and B cells. By summarizing key findings and discussing future directions, this concise review aims to elucidate the complex interplay between immune cells and fibrosis in IPF, offering insights that could guide novel therapeutic strategies in managing this debilitating disease.

## 2. Evidence of Immune Dysregulation in Patients with Idiopathic Pulmonary Fibrosis (IPF)

At its foundation, IPF is thought as a disrupted or uncontrolled wound-healing process in the lung, leading to excessive scar formation and progressive lung dysfunction. There is substantial evidence from various perspectives that the immune system plays a role in the development and progression of IPF.

### 2.1. Infiltration of Immune Cells around Fibroblast Foci

As stated in the 2022 ATS guideline, a diagnosis of UIP, which is the hallmark pathology of IPF, made by biopsy is predicated on a combination of the following: (1) patchy dense fibrosis with architectural distortion (*i.e*., destructive scarring and/or honeycombing); (2) a predilection for subpleural and paraseptal lung parenchyma; (3) fibroblast foci; and (4) the absence of features that suggest an alternative diagnosis [1]. Spatial transcriptomics used to deconstruct the cellular composition of IPF lungs has revealed a stepwise increase in the number of proliferating macrophages as proximity to fibrotic sites decreased. These macrophages were primarily located in distant alveolar septae, with their highest concentration in regions adjacent to fibroblastic foci, but were notably absent from the stiffened stroma within the fibroblastic foci itself [2]. In end-stage lung explants taken during lung transplantation, lymphocyte aggregates, primarily consisting of CD3+ T lymphocytes and CD20+ B lymphocytes, are found to increase significantly compared to those in surgical lung biopsy samples representing early disease stages [3]. These immune cells exhibit minimal or no activity of Ki-67, a marker for cell proliferation, and Caspase-3, a marker for apoptosis, suggesting that they are not proliferating locally within the tissue but are instead likely recruited from the bloodstream.

### 2.2. Serology Markers in IPF Patients

Serologically, multiple markers are associated with the diagnosis and prognosis of the disease. For example, Todd et al. utilized 300 patients with IPF from the IPF-PRO Registry, comparing them with 100 control participants to identify differentially expressed proteins and correlate these with disease severity [4]. Several immune-related proteins were elevated in IPF patients, including chemokines such as CCL5, CCL17, CCL18, CCL22, and CXCL13, as well as complement proteins (C1R, C4A, and C4B). CXCL13 is significantly elevated in both the plasma and lungs of IPF patients and correlates with disease severity and poor prognosis. Patients with the highest CXCL13 levels had a significantly higher risk of death or requiring emergent lung transplantation within two years [5].

### 2.3. Genetic Polymorphisms and Differential Gene Expression of Immune Cells in IPF

Gene polymorphism involving inflammatory regulators may be associated with pulmonary fibrosis [6], which includes IL-1RN [7], IL-4 [8], Toll-like receptor 3 [9], and TOLLIP (Toll-interacting protein) [10] among others. The prognosis of IPF has also been linked with specific genetic expression profiles of peripheral blood mononuclear cells (PBMC). In their first experiment, Herazo-Maya et al. performed microarray analysis of the PBMC on two IPF patient cohorts: a discovery cohort (*n* = 45) from the University of Chicago and a replication cohort (*n* = 75) from the University of Pittsburgh. They identified 52 genes significantly associated with transplant free survival (TFS) [11]. This was subsequently validated across six independent cohorts from academic centers in the United States, United Kingdom, and Germany, enrolling 425 IPF patients [12]. The expression levels of the 52 genes were assessed, and two scores were calculated: an “up score” and a “down score” based on the expression levels of genes. Patients whose up score was above the median and down score below the median were classified as high risk. In all cohorts, high-risk patients had a higher mortality rate or shorter TFS compared to low-risk patients. The hazard ratios (HR) for mortality and TFS ranged from 2.03 to 4.37 across the different cohorts, indicating that high-risk patients had at least double the risk of death or lung transplantation. Furthermore, in patients not treated with anti-fibrotic drugs, there was no significant change in the scores over time, suggesting that untreated patients maintain their risk profile [12]. They retain their discrimination even when treated with antifibrotics [13]. Further classification of these genes revealed that the seven genes upregulated were primarily expressed by monocytes, while the 45 genes that were downregulated were primarily expressed by T, B, and NK cells [14].

## 3. History of Immunosuppression Treatment in IPF Patients

As would be expected with this evidence, the treatment of IPF primarily used to aim at suppressing inflammation. The 2000 ATS/ERS guidelines, while acknowledging the poor prognosis of IPF and the lack of evidence showing improved survival with any treatment, recommended the use of corticosteroids combined with either azathioprine or cyclophosphamide as initial therapy [15]. However, the guidelines also cautioned that the potential benefits of these treatments could be outweighed by the risks of treatment-related complications. As oxidative stress was hypothesized to contribute to epithelial injury in IPF, antioxidant therapies were explored as potential treatment options. N-acetylcysteine (NAC), a precursor to the antioxidant glutathione, was proposed as an adjunct to immunosuppressive therapy for IPF patients.

The 2005 IFIGENIA trial, published in the New England Journal of Medicine, suggested that adding high-dose NAC to prednisone and azathioprine helped preserve vital capacity and lung diffusion in IPF patients [16]. However, this trial faced criticism for lacking a true placebo group, and the efficacy of the treatment regimen remained contentious, though it became a common practice at the time. A survey conducted between December 2006 and January 2007 revealed that nearly 50% of the 800 pulmonologists who responded would prescribe a combination of prednisone and azathioprine, with or without NAC in two hypothetical cases of IPF [17]. A more definitive understanding of the use of broad immunosuppression in IPF came with the PANTHER-IPF trial [18], a randomized, double-blind, placebo-controlled study that compared the effects of prednisone, azathioprine, and NAC (combination therapy) to NAC alone and placebo in IPF patients with mild-to-moderate lung function impairment. The primary outcome was the change in forced vital capacity (FVC) over a 60-week period. An interim analysis at 32 weeks revealed a higher rate of death and hospitalization in the combination therapy group compared to placebo. Based on these findings, the independent data and safety monitoring board recommended discontinuing the combination therapy group. This has led to a significant shift away from broad immunosuppressive treatments in IPF.

Following this paradigm shift, various attempts to explore immunomodulatory therapies in IPF failed to meet their primary endpoints, which often included changes in FVC, disease progression, or survival. These trials included studies investigating interferon-gamma (INSPIRE study) [19], TNF-alpha inhibitors [20], NAC monotherapy [21], monoclonal antibodies targeting inflammatory cytokine IL-13 [22,23], CCL2 [24] and dual inhibition of IL-13 and IL-4 [25]. Despite these efforts, no immunomodulatory treatment has demonstrated significant clinical benefit in IPF, further moving the focus of IPF management away from immunosuppressive strategies. However, these drugs do not target specific immune cell populations, and it is possible that the manipulation of specific immune cell subsets could be a viable therapeutic approach in IPF.

## 4. Innate Immune System

### 4.1. Monocyte/Macrophage

Several studies involving IPF patients have implicated macrophages and monocytes in the pathogenesis and prognosis of the disease. Transcriptome data from peripheral blood mononuclear cell samples of IPF patients revealed that monocyte percentage above the mean was linked to shorter transplant-free survival [26]. This finding was further validated in a retrospective pooled analysis of four phase III randomized trials [27], involving 2067 patients from the ASCEND [28] and CAPACITY trial [29], which studied pirfenidone, and the INSPIRE trial [19], which evaluated interferon-gamma. Elevated monocyte percentages were significantly associated with one-year IPF progression (defined as a >10% absolute decline in FVC% predicted, a >50 m decline in six-minute walk distance, or death), as well as with increased risk of one-year all-cause hospitalization and mortality. Similar findings were observed in a systematic review and meta-analysis of patients with fibrotic ILD, as well as in a study involving patients with interstitial lung abnormalities detected through imaging [30,31]. In accordance, several studies have shown that mice with systemic monocytopenia are protected from the development of fibrosis. This protection has been observed in mice treated with intravenous liposomal clodronate, as well as in genetically modified CCR2−/− and CEBPd−/− knockout mice, which lack specific monocyte populations [32–34]. These findings suggest that circulating monocytes play a significant role in the fibrotic process and that their depletion or functional disruption can mitigate fibrosis development.

#### 4.1.1. Tissue Resident Macrophages *vs*. Recruited Macrophages in Mouse Models of Pulmonary Fibrosis

Alveolar macrophages (AMs) constitute the primary immune defense within the alveoli and airways, whereas lung interstitial macrophages (IMs) serve as pivotal regulators of the vasculature and lung interstitium. Both AMs and IMs are categorized as tissue-resident macrophages (TRMs), performing critical functions in maintaining homeostasis, facilitating metabolic processes, and mediating tissue repair in their respective organ environments. Additionally, these macrophages function as sentinel phagocytes within the immune system. During inflammatory conditions, monocytes are recruited to the lung, where they differentiate into recruited macrophages, exhibiting distinct transcriptional profiles and specialized functional roles [35]. Macrophage populations within tissues, derived from unique developmental lineages, express diverse surface markers that reflect their lineage and functional specificity. Classical monocytes are identified by the combination of MHC II^negative^, CD64^low^, CD11b^high^, and Ly6C^high^. Tissue resident interstitial macrophages are characterized by high expression of MHC II, CD64, CD11b, while being negative for Siglec F [35,36]. These macrophages can be further classified into subpopulations with distinct functional roles. Some are involved in maintaining tissue homeostasis and regulating vascular permeability, while others participate in immune surveillance and pro-inflammatory responses. The differentiation of these subpopulations is determined by surface markers such as LYVE-1 (lymphatic vessel endothelial hyaluronan receptor-1), Folr2 (folate receptor beta), CD206 (mannose receptor), Arg1 (arginase-1), and CX3CR1 [37,38]. Tissue-resident alveolar macrophages are identified by CD64^high^, CD11c^high^, F4/80 positive, MerTK positive, and Siglec F^high^ [36].Monocyte-derived macrophages are identified by CD64^high^, F4/80 positive, MerTK positive, and Siglec F^low^. During differentiation from monocyte-derived interstitial macrophages to monocyte-derived alveolar macrophages, they exhibit a decrease in CD11b and an increase in CD11c [35].Using various fate-mapping techniques, several groups have demonstrated that in naïve, unchallenged adult mice housed in clean facilities, tissue-resident alveolar macrophages maintain their population via proliferation in situ for months without contribution from circulating monocytes [39,40].Similarly, two clinical reports revealed that after lung transplantation, the alveolar macrophage population remains remarkably stable, with donor-derived cells making up the majority of the alveolar macrophage pool even five years post-transplant [41,42]. Although in the healthy, unperturbed lung at steady state, the origin of alveolar macrophages—whether from fetal or adult sources or from differentiated alveolar macrophages—does not influence their ability to occupy the lung niche [43,44], tissue-resident macrophages and monocyte-derived macrophages frequently display distinct, sometimes opposite, roles in various injury models, including cardiac injury [45], schistosomiasis (a parasite infection) [46].

Evidence suggested that tissue-resident alveolar macrophages (TR-AMs) are not actively involved in the fibrotic response, whereas monocyte-derived alveolar macrophages (Mo-AM) play a more significant role. In a study conducted by Gibbons et al., the depletion of circulating monocytes during the fibrotic phase of bleomycin-induced lung fibrosis resulted in reduced fibrosis [32]. Specifically, the depletion of the “inflammatory” Ly6C^high^ monocyte subset significantly decreased the number of Ym1-positive alternatively activated macrophages in the lungs. The adoptive transfer of Ly6C^high^ monocytes into bleomycin-treated mice during the fibrotic phase exacerbated lung fibrosis, as evidenced by increased collagen deposition and a higher number of Ym1-positive macrophages. Using bone marrow chimera mice, Misharin et al. demonstrated that in bleomycin-induced lung fibrosis, circulating monocytes are recruited to the lung and differentiate into Mo-AMs [47]. By utilizing CD11cCre Casp8flox/flox and LysMCre Casp8flox/flox mice to selectively deplete caspase-8, a suppressor of necroptosis, to markedly reduce the Mo-AM population, fibrosis was attenuated. In contrast, the depletion of TR-AMs using intratracheal liposomal clodronate did not affect fibrosis severity. Using similar CD11cCreCasp8flox/flox mice in an asbestos induced lung fibrosis model, Joshi et al. further demonstrated monocyte-derived alveolar macrophages were specifically located in these fibrotic areas, around bronchoalveolar duct junctions where asbestos fibers lodged, where they co-localized with fibroblasts, forming a fibrotic niche [48]. These macrophages expressed high levels of markers associated with fibrosis, such as Csf1, Pdgfa, and Mrc1, supporting their role in driving fibrosis through their interactions with fibroblasts. A different strategy to study the contribution of Mo-AMs in pulmonary fibrosis was used by McCubbrey et al. [49]. Their approach used hCD68rtTAcre (reverse tetracycline-controlled transactivator) floxed c-FILP (cellular FADD-like IL-1β–converting enzyme–inhibitory protein) which allowed for the inducible deletion of c-FLIP specifically in CD11b^high^ macrophages, making them susceptible to apoptosis upon administration of doxycycline. The study found that conditional deletion of c-FLIP in CD11b^high^ macrophages resulted in their significant depletion from the lung protecting the mice from developing lung fibrosis as reflected in the histology and lung compliance.

**Figure 1.**
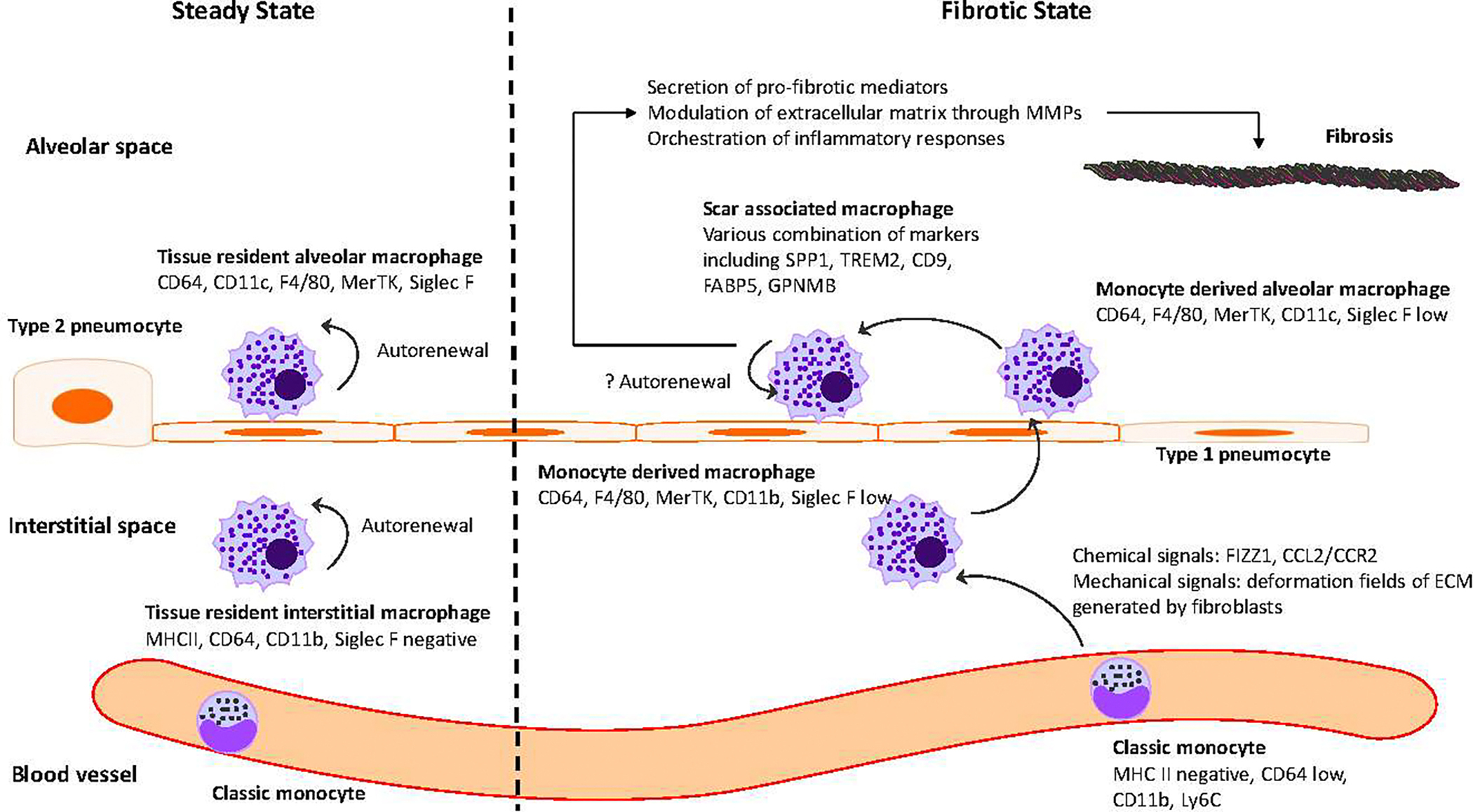
Monocytes and macrophages in steady state and fibrosis in mouse pulmonary fibrosis model. Monocytes and macrophages exhibit a diverse range of surface markers that reflect their lineage and functional specialization. In steady state, tissue-resident macrophages (alveolar and interstitial) maintain homeostasis via self-renewal. They can be identified by surface markers, including CD64^high^, CD11c^high^, F4/80 positive, MerTK positive, Siglec F^high^ for alveolar macrophages and MHC II^high^, CD64^high^, CD11b^high^, Siglec F^negative^ for interstitial macrophages, respectively. In response to chemical and mechanical cues, monocyte-derived macrophages, recruited under fibrotic conditions, exhibit distinct marker profiles CD64^high^, F4/80^positive^, MerTK^positive^, and Siglec F^low^. As monocyte-derived interstitial macrophages differentiate into monocyte-derived alveolar macrophages, they exhibit a decrease in CD11b expression and an increase in CD11c expression. These macrophages can further transition into scar-associated macrophages, characterized by variable expression of markers such as SPP1, TREM2, CD9, FABP5, and GPNMB. Scar-associated macrophages contribute to fibrosis through a range of direct mechanisms, such as extracellular matrix modulation, and indirect mechanisms, such as immune response orchestration.

In summary, these studies collectively demonstrate the pivotal role that monocyte derived macrophage populations play in the development and progression of lung fibrosis. A summary of monocyte and macrophage dynamics in steady-state and fibrosis in the mouse pulmonary fibrosis model is illustrated in Figure 1.

#### 4.1.2. Macrophage Heterogeneity in IPF

##### The Classic M1/M2 Polarization Concept

Given the significant overlap in surface marker expression between different macrophage subsets, an effective approach to their characterization has been the analysis of specific gene expression profiles following cytokine or microbial stimulation. Classically activated macrophages (M1) are primarily involved in host defense mechanisms against bacteria, protozoa, and viruses, and they also play a key role in antitumor immunity. In contrast, alternatively activated macrophages (M2) are known for their anti-inflammatory properties and their contributions to tissue repair and wound healing [50]. Notably, using surgical lung biopsies, studies have demonstrated that usual interstitial pneumonia is associated with significantly higher levels of IL-13 and its receptor subunits, IL-13Ra2 and IL-13Ra1, particularly in fibroblastic foci [51,52]. Given their role in tissue remodeling, fibrosis has traditionally been considered an M2-dominated disease.

In experimental models of lung fibrosis, various therapeutic interventions have focused on reducing the presence or activity of M2 macrophages. These interventions include targeting TNF-alpha [53], inhibiting Gab1 and Gab2 adaptor proteins, which are key players in the IL-4 signaling pathway [7], and neutralizing IL-33 [54]. Additionally, the use of microcystin-leucine arginine (microcystin-LR), an environmental cyanobacterial toxin, in bleomycin-induced pulmonary fibrosis models, and serum amyloid P (SAP) in TGF-beta overexpression models have also shown efficacy in reducing lung fibrosis [55].

Despite the encouraging results from these preclinical studies that aimed to interfere with M2 macrophages, clinical phase III trials in humans have largely yielded disappointing outcomes. One possible explanation for these results is that macrophage polarization and immune cell differentiation in the disease state are not mutually exclusive or as well-defined as in healthy conditions. Indeed, research has shown that monocytes isolated from the peripheral blood of IPF patients exhibit a significantly upregulated Type I interferon (IFN) response, marked by the overexpression of interferon-stimulated genes such as MX1, ISG15, and OASL [56]. Additionally, resident memory T cells from explanted lungs of IPF patients have been found to exhibit IFN-γ–mediated responses, further complicating the immune landscape in IPF [57]. It is important to note that the decreased production of certain cytokines or the presence of skewed immune cell populations in fibrotic lungs may represent an altered lung microenvironment rather than an inherent defect in cytokine production. Consequently, the administration of exogenous cytokines alone has proven insufficient to fully re-differentiate immune cells in the fibrotic milieu [58]. This suggests that fibrosis involves a complex interplay of immune cells and signaling pathways that cannot be corrected by targeting a single cytokine or cell type, highlighting the need for more comprehensive therapeutic strategies.

##### The Pathological Macrophage Subsets in IPF Revealed by High-Dimensional Transcriptomics

Single-cell RNA sequencing has revealed multiple distinct macrophage populations in the lung tissue of IPF patients, including a recruited macrophage subpopulation characterized by high expression of SPP1 (osteopontin) and CHIT1 (chitinase 1) [59]. In healthy lungs, FABP4^high^ macrophages constitute the predominant alveolar macrophage subpopulation, while SPP1^high^ macrophages are present at low levels. However, in fibrotic regions of IPF lungs, the expansion of SPP1^high^ macrophages, accompanied by a reduction in FABP4^high^ macrophages, particularly in the lower lung lobes, correlates with poor lung function, as evidenced by lower forced vital capacity percentage predicted (FVC% pred) [60]. Trajectory analysis indicates that these SPP1^high^ macrophages originate from circulating CD14+ monocytes, not tissue-resident macrophages, underscoring their role as recruited monocyte-derived macrophages central to the fibrotic process in IPF [59].

In fact, studies across disease models and species identify pathological macrophage subsets localized near ECM-producing fibroblasts, commonly referred to as scar-associated macrophages (SAMs), fibrosis-associated macrophages, or matrisome-associated macrophages. These subsets, which commonly express SPP1, TREM2, CD9, FABP5 (Fatty Acid Binding Protein 5), and GPNMB (Glycoprotein Non-Metastatic B), are implicated in various fibrotic diseases other than pulmonary fibrosis, including mouse models of skeletal muscle fibrosis [61], metabolic-associated fatty liver disease/nonalcoholic steatohepatitis [62–64], myocardial infarction [65], ureteric obstruction models [66], and human conditions such as acne keloidalis [67], liver cirrhosis [68,69], nonalcoholic fatty liver disease/steatohepatitis [70,71], post-COVID fibrosis [72], dilated cardiomyopathy [73], and myocardial infarction [74]. These findings underscore the conserved role of these macrophages in fibrotic diseases across tissues and species, highlighting them as potential therapeutic targets in fibrosis modulation.

#### 4.1.3. Macrophage Recruitment

Macrophage recruitment to sites of tissue injury and fibrosis is a complex process influenced by various chemical and mechanical signals.

##### Chemical Signals

One important chemoattractant identified in macrophage recruitment is FIZZ1 (Found in Inflammatory Zone 1). According to migration assays, FIZZ1 has been shown to possess chemoattractant activity for bone marrow-derived cells, including macrophages. Studies have demonstrated that bone marrow cells from both PBS- and bleomycin-treated mice migrated toward FIZZ1, although the response was stronger in control mice. *In vivo* experiments further reinforced the importance of FIZZ1 in fibrosis. FIZZ1 knockout mice exhibited significantly reduced recruitment of bone marrow cells to the lungs following bleomycin treatment. This reduction in bone marrow cell recruitment also correlated with lower numbers of inflammatory cells, including macrophages, in the bronchoalveolar lavage fluid, compared to wild-type mice [75].

Another important signaling pathway involved in macrophage recruitment is the CCL2/CCR2 axis. CCL2 is a potent chemoattractant responsible for the recruitment of fibrocytes and profibrotic macrophages to sites of tissue injury. Elevated levels of CCL2 have been identified in patients with idiopathic pulmonary fibrosis, highlighting its role in fibrosis progression [56]. In mouse models, the disruption of this signaling pathway has provided protection against lung fibrosis. For instance, CCR2 knockout mice were shown to be protected from lung fibrosis induced by bleomycin treatment [33,76] and intrathecal instillation of FITC (Fluorescein Isothiocyanate) [77,78]. These findings suggest that inhibiting the CCL2/CCR2 axis could be a potential therapeutic target in preventing fibrosis.

However, the translation of these findings into human therapies has proven to be more complex. For instance, a phase 2 randomized, double-blind placebo-controlled trial using carlumab, a human immunoglobulin G1κ monoclonal antibody designed to specifically neutralize the profibrotic activities of CCL2, demonstrated an unexpected outcome. Patients treated with carlumab showed a decline in forced vital capacity (FVC), a key indicator of lung function. Moreover, contrary to expectations, both total and free CCL2 levels were found to be elevated in the treated patients [24]. This paradoxical increase suggests that compensatory mechanisms may come into play upon blockade of the CCL2/CCR2 pathway. In line with this observation, a CCL12 knockout mouse model—where CCL12 is a murine homolog of CCL2—showed a compensatory increase in the expression of other CCR2 ligands, including CCL2 and CCL7, and did not exhibit protection from fibrosis following bleomycin treatment [79]. These findings underscore the complexity of chemokine signaling in fibrosis and highlight the potential limitations of targeting individual pathways without addressing compensatory mechanisms.

##### Mechanical Signals

In addition to chemical signals, mechanical cues in the extracellular matrix (ECM) have emerged as critical regulators of macrophage morphology, migration, activation, and function. Surface topographic features and stiffness significantly influence macrophage behavior and have been extensively studied in the context of foreign body reactions [80–82].

The mechanical properties of the fibrotic lung undergo significant changes during fibrosis progression. These include increased tissue stiffness (with healthy lung tissue having a Young’s modulus of approximately 1.96 kPa compared to up to 16.5 kPa in fibrotic lungs), altered viscoelastic properties, elevated surface tension, and regional heterogeneity [83]. In addition, the fibrotic ECM is characterized by excessive fibrillar collagen aligned in dense bundles, a result of active ECM remodeling by contractile myofibroblasts. These structural changes provide macrophages with additional topographic and mechanical cues that modulate their behavior [84].

Xu et al. explored how macrophages prepolarized with IL-4/13 respond to such cues in pulmonary fibrosis. Using a collagen hydrogel system with varying stiffness and fiber alignment, the authors demonstrated that macrophages in regions of high ECM stiffness or strong collagen alignment exhibited elongated morphologies and aligned along collagen fibers, displaying enhanced migratory behavior. These macrophages polarized into a pro-fibrotic phenotype, characterized by increased secretion of transforming growth factor-beta (TGF-β) and interleukin-6 (IL-6). Mechanistically, this response was mediated through integrin αMβ2 and cytoskeletal remodeling driven by Rho-associated kinase 2 (ROCK2). The process was shown to be inhibited by pirfenidone, an FDA-approved anti-fibrotic drug, which disrupts integrin signaling and cytoskeletal reorganization [84].

Beyond static mechanical cues, the dynamic interplay between macrophages and fibroblasts plays an important role in regulating macrophage motility and phenotype. In dense collagen networks (DCNs), macrophages relied on tunnel-like conduits created by fibroblasts to migrate through the ECM. In loosely connected networks (LCNs), macrophages utilized fibroblast-aligned collagen fibers as directional tracks for movement [85].The mechanical remodeling of the ECM by fibroblasts not only facilitated macrophage migration but also guided their positioning in fibrotic regions.

Dynamic mechanical cues, such as deformation fields generated by fibroblast contractions, further amplify macrophage recruitment. A study by Pakshir et al. demonstrated that macrophages detect the velocity of local substrate displacements and migrate toward regions of force generation, even in the absence of chemotactic gradients. This mechanosensing process, facilitated by integrin α2β1 and stretch-activated ion channels, highlights the sophisticated mechanical communication between contractile fibroblasts and macrophages. Importantly, dynamic forces were essential for macrophage migration, as static ECM cues alone, such as collagen alignment, were insufficient to drive their movement [86].

However, not all studies reach the same conclusions regarding macrophage mechanosensing in fibrotic environments. Using 3D collagen and polyacrylamide (PA) hydrogels to decouple ECM stiffness from collagen concentration, another study revealed that macrophages exhibit differential gene expression based on ECM stiffness. For example, the expression of Fizz1, a gene associated with tissue repair, was suppressed in stiffer ECM environments, while Arg1 expression remained unaffected. Unlike the integrin-dependent mechanisms described in previous studies, this process relied on cytoskeletal remodeling, which regulated chromatin accessibility to control mechanosensitive gene expression. Similar results are replicated *in vivo* using a bleomycin-induced pulmonary fibrosis model. The authors conclude that these findings suggest that macrophage sensing of ECM mechanics could provide a negative feedback mechanism to prevent excessive ECM deposition and maintain homeostasis [87].

Although these conclusions may initially appear contradictory, the differences likely stem from variations in experimental conditions, such as the biomechanical properties of substrates (e.g., differing thresholds of stiffness), architectural features (e.g., two-dimensional versus three-dimensional structures, or specific topographic characteristics), and the involvement of fibroblasts. Furthermore, these discrepancies underscore the complex and multifaceted role of macrophages in fibrosis and highlight the inherently resolving nature of the mouse bleomycin-induced pulmonary fibrosis model. Together, these findings emphasize the nuanced interplay of macrophages with mechanical and dynamic cues in the fibrotic microenvironment, offering insights into potential therapeutic strategies for modulating macrophage activity in fibrosis.

#### 4.1.4. Macrophage Effects

Once recruited, macrophages are able to influence fibrosis by multiple mechanisms: secretion of pro-fibrotic mediators, including TGF-beta [58], directly modulating extracellular matrix through matrix metalloproteinases (MMPs) [88,89] and indirectly orchestrating inflammatory responses [90].

#### 4.1.5. Fate of Macrophages

Survival of macrophages is contingent on specific microenvironmental cues and cellular interactions. Tissue-resident alveolar macrophages depend on granulocyte-macrophage colony-stimulating factor (GM-CSF), which is primarily produced by alveolar type II (ATII) cells, while monocyte derived macrophages rely on macrophage colony-stimulating factor (M-CSF) for their maintenance [91–93]. The fate of monocyte-derived macrophages in the context of pulmonary fibrosis has been the subject of considerable research, with varying results across different models. Studies have reported divergent lifespans for these macrophages, ranging from less than 24 h [32] to up to a year [47].

The maintenance of macrophage populations may occur through either autocrine or paracrine signaling mechanisms, wherein cells either self-sustain by secreting their own survival factors or rely on signals from neighboring cells within the microenvironment. Recent studies have provided further insights. These analyses utilizing single-cell RNA sequencing and in situ RNA hybridization revealed that M-CSF receptor (M-CSFR) and its ligand, M-CSF, are upregulated in fibrotic macrophages. The detection of CSF1/CSF1R expression in alveolar macrophages suggests that these cells may be capable of maintaining their population in the fibrotic niche via autocrine production of M-CSF, thus becoming independent of external signals from other lung cells [48]. This autocrine loop likely contributes to the persistence of macrophages in the fibrotic lung, which in turn sustains fibrosis by continually promoting ECM production and remodeling. In a computational model exploring the interaction between macrophages and fibroblasts, researchers further elucidated the importance of M-CSF in sustaining the fibrotic process. The model demonstrated that reducing CSF1 levels led to a decrease in macrophage numbers, which, in turn, resulted in a reduction in fibroblast activity. This allowed the tissue to transition toward a more normal, less fibrotic state [94].

Interruption of this M-CSF/M-CSFR signaling pathway has shown therapeutic promise in preclinical models. Blockade of this signaling using an anti-M-CSF antibody or the small molecule PLX3397 led to a significant reduction in monocyte-derived macrophages and subsequently ameliorated fibrosis [48]. This finding highlights the potential of targeting macrophage survival, by targeting key signaling pathways, such as the M-CSF/M-CSFR axis, as a therapeutic strategy for fibrosis.

#### 4.1.6. Therapeutics Targeting Macrophages

Due to the pivotal role macrophages play in the progression of pulmonary fibrosis, targeted therapies focusing on these cells are highly desired. Of these approaches, nanoparticles and liposomes are among the most widely explored platforms for delivering therapeutic agents directly to macrophages. Their versatility lies in their ability to encapsulate diverse payloads, including small molecules, siRNA, mRNA, and proteins, which can modulate macrophage behavior effectively. To increase specificity, these carriers can be further engineered by modifying their surfaces with ligands or antibodies targeting macrophage-specific markers, such as CD206, a receptor highly expressed on M2 macrophages in fibrotic tissues.

In a notable study, Singh et al. engineered mannosylated albumin nanoparticles (MANPs) to exploit the high expression of the mannose receptor (CD206) on profibrotic monocyte-derived macrophages. Using a bleomycin-induced pulmonary fibrosis mouse model, the researchers demonstrated that approximately 75% of the injected MANPs were taken up by monocyte-derived alveolar macrophages. This was confirmed using Cx3cr1CreERT2+, Tdtomato fl/fl genetic lineage tracing mice. To further explore therapeutic potential, MANPs were loaded with small-interfering RNA (siRNA) targeting TGFβ1. Treatment with MANPs significantly reduced collagen deposition and other fibrotic markers, including TGFβ1 and IL-1β, while improving lung function. These findings highlight the efficacy of macrophage-targeting nanoparticles in mitigating fibrosis and restoring lung architecture [95]. Similar results were reported by Hou et al., who utilized M2pep-modified Mn-curcumin metal-organic framework nanoparticles (M2NP-BLZ@Mn-Cur). These nanoparticles feature a Mn-curcumin metal-organic framework coupled with M2pep ligands to enhance targeting specificity toward M2 macrophages, and BLZ945, a CSF-1R inhibitor, to facilitate the selective depletion of profibrotic M2 macrophages [96].

Although studies specifically focusing on nanoparticle-mediated macrophage targeting for pulmonary fibrosis are limited, lessons can be drawn from their application in other diseases, such as atherosclerosis. Nanoparticles have been extensively explored in the treatment and diagnosis of atherosclerosis, with multiple clinical trials underway. As summarized in a review, macrophage-targeting nanoparticles in atherosclerosis have been employed to inhibit monocyte recruitment, suppress macrophage proliferation, restore efferocytosis, inhibit inflammation, and induce macrophage apoptosis [97]. These therapeutic mechanisms are highly relevant to pulmonary fibrosis.

Folate receptor-beta (FR-β), a glycosylphosphatidylinositol-anchored glycoprotein, represents another promising macrophage-specific target. FR-β mediates the unidirectional transport of folate into cells and is overexpressed on activated macrophages, while its expression is undetectable on resting macrophages [98]. Researchers have utilized this receptor to deliver therapeutic agents selectively to macrophages in fibrotic tissues. In one example, a folate-linked TLR7 agonist (FA-TLR7-54) was developed to reprogram profibrotic macrophages. When administered intravenously to bleomycin-treated mice, FA-TLR7-54 predominantly accumulated in the lungs. Flow cytometry confirmed that the nanoparticles were selectively taken up by FR-β-positive macrophages, primarily monocyte-derived alveolar macrophages. FA-TLR7-54 effectively shifted macrophages from an M2-like profibrotic phenotype to an M1-like antifibrotic state. This polarization shift resulted in the suppression of profibrotic cytokines such as CCL18, IL-1β, and Arg1, while promoting antifibrotic markers like CXCL10, IL-6, and IFNγ. Notably, the treatment reduced fibrosis without inducing significant systemic toxicity, as evidenced by stable body weight, normal plasma cytokine levels, and unaffected histology in major organs [99].

Elevated miR-33 levels in bronchoalveolar lavage (BAL) cells and lung macrophages of idiopathic pulmonary fibrosis (IPF) patients suggest its involvement in disease progression. Ahangari et al. developed a peptide nucleic acid (PNA)-based miR-33 inhibitor (PNA-33) that can be delivered both intranasally and intravenously. While intranasal delivery was more efficient, systemic administration also showed therapeutic benefits. Pharmacological inhibition of miR-33 with PNA-33 improved mitochondrial homeostasis and enhanced autophagy in macrophages. These effects translated into significant attenuation of fibrosis in bleomycin-induced pulmonary fibrosis mouse models. The study underscores the importance of targeting macrophage metabolic pathways and mitochondrial function to mitigate fibrotic progression [100].

While each of these strategies focuses on distinct molecular pathways, they collectively highlight the promise of macrophage-targeted therapies in addressing pulmonary fibrosis by offering high precision and efficacy while minimizing systemic toxicity. The ability to reprogram macrophages from a profibrotic to an antifibrotic state provides a multifaceted approach to tackling this complex disease. Future studies should build on these findings to optimize delivery systems, explore combination therapies, and translate these promising preclinical results into clinical applications.

### 4.2. Innate Lymphoid Cells

Innate lymphoid cells (ILCs) are a recently identified group of immune cells that, unlike T and B lymphocytes, do not express recombination-activating genes, which are responsible for the somatic recombination of antigen receptors. ILCs are classified into three major subsets: ILC1, ILC2, and ILC3, based on their cytokine production profiles and transcription factor dependencies, paralleling the functional characteristics of CD4+ T helper cell subsets [101]. While the role of ILCs in immune responses is relatively underexplored compared to other lymphocytes, growing evidence points to the significant involvement of group 2 innate lymphoid cells (ILC2s) in the pathogenesis of pulmonary fibrosis.

ILC2s are primarily activated by a group of epithelial-derived cytokines known as “alarmins”, which include IL-25, IL-33, and thymic stromal lymphopoietin (TSLP) [101]. Once activated, ILC2s secrete large amounts of type 2 cytokines, including IL-5, IL-9, and IL-13 [102]. These cytokines are crucial mediators of fibrotic responses, as mentioned above in the M1/M2 polarization and Th1/Th2 cell section.

Experimental models using bleomycin-induced pulmonary fibrosis have provided evidence for the involvement of ILC2s in fibrotic processes. Mice genetically deficient in ILC2s, such as staggerer mice (Rorasg/sg) that lack the transcription factor RORα essential for ILC2 development [103], as well as mice deficient in the IL-33 receptor ST2 [104], exhibit significantly reduced fibrosis compared to wild-type controls. However, despite these promising findings, the precise mechanisms by which ILC2s contribute to fibrosis remain incompletely understood, necessitating further studies to elucidate the complex signaling pathways involved and to explore potential therapeutic strategies targeting ILC2s or their upstream activators in fibrotic lung diseases.

## 5. Adaptive Immune System

### 5.1. T Helper (Th) Cells

#### 5.1.1. Th1/Th2 Balance

CD4+ T cells are traditionally classified into two main subtypes, Th1 and Th2, based on their cytokine expression profiles and their distinct functional roles in immune responses. Th1 cytokines primarily include IL-2, IFN-γ, tumor necrosis factor, IL-12, and IL-18, while Th2 cytokines predominantly comprise IL-4, IL-5, IL-6, IL-10, IL-13, and monocyte chemotactic protein-1 [105]. Generally, Th1 cells are regarded as anti-fibrotic, whereas Th2 cells are associated with pro-fibrotic activities. This dichotomy has been demonstrated in patients with IPF and various *in vivo* and *in vitro* models. Early studies have shown a reduction in IFN-γ and an increase in IL-4 levels in the bronchoalveolar lavage and serum of patients with fibrosis, previously referred to as cryptogenic fibrosing alveolitis [106–108]. Additionally, polymorphisms in the IL-4 promoter region have been linked to IPF [109]. Genetic knockout of T-bet, a key Th1 transcription factor, results in an increase in fibrosis after bleomycin treatment, with a corresponding rise in Th2 cytokines and TGF-β [110]. Conversely, overexpression of GATA-3, a Th2 transcription factor, leads to similar outcomes [111]. Despite these findings, efforts to restore the Th1/Th2 balance in therapeutic settings have not translated into meaningful clinical benefits.

#### 5.1.2. Th17 Cells

Type 17 immunity has also been implicated in the pathogenesis of IPF [112]. IL-17, a key cytokine produced by Th17 cells, has several functions, including the stimulation of extracellular matrix production, promotion of collagen deposition, and mediation of TGF-β signaling [102]. Studies have shown that CD4+ T cells from IPF patients exhibit significantly higher levels of PD-1 expression, particularly within the Th17 subset. This upregulation is associated with increased production of IL-17A and TGF-β1, leading to enhanced collagen production by human lung fibroblasts in co-culture experiments. In murine models of bleomycin-induced fibrosis, targeting PD-1, either through genetic knockout or with an anti-PD-1 antibody, resulted in reduced lung fibrosis [113]. Additionally, direct targeting of Th17 cells by IL-17A knockout or the use of an anti-IL-17A antibody has been shown to decrease fibrosis [114,115]. Similarly, administration of IL-27, which suppresses Th17 differentiation, also reduced fibrosis in these models [116].

#### 5.1.3. Treg Cells

Regulatory T cells (Tregs) play a dual role in fibrosis due to their ability to produce both IL-10 and TGF-β1, giving them the capacity to either promote or suppress fibrosis depending on the context [102]. However, the precise role of Tregs in IPF remains controversial, as studies have yielded inconsistent results. Early research demonstrated a decrease in CD4 + CD25 + FOXP3+ Tregs in both the peripheral blood and BAL of IPF patients, coupled with a reduced ability to suppress effector T cell proliferation [117]. In contrast, a study by Reilkoff et al. found that a distinct subset of Tregs expressing Semaphorin-7a was significantly increased in the lungs and blood of IPF patients when compared to controls [118]. Further investigation by Unterman et al. using single-cell RNA sequencing to analyze peripheral blood mononuclear cells from IPF patients provided additional insight into the variability of Tregs across different stages of the disease [14]. In a cohort of 25 patients categorized as either stable or progressive based on 36-month all-cause mortality, Tregs were found to be reduced in stable IPF patients compared to those with progressive disease and healthy individuals. Notably, when considering only T cells, Treg numbers were elevated in patients with progressive disease, suggesting a dynamic role for Tregs that may depend on disease progression and phenotype. In animal models, adoptive transfer experiments have demonstrated that Tregs expressing Semaphorin-7a can actively induce fibrosis when transferred into TGF-β1-expressing mice, further supporting the hypothesis that certain Treg subsets may contribute to the fibrotic process [118]. A study by Boveda-Ruiz explored the timing of Treg depletion in a bleomycin-induced lung fibrosis model using an anti-CD25 antibody [119]. The results showed that early depletion of Tregs, prior to bleomycin infusion, led to a significant reduction in lung inflammation, collagen deposition, and overall fibrosis. In contrast, depletion of Tregs during the late phase of fibrosis exacerbated the disease. The study also indicated that Treg depletion at different stages of fibrosis influenced the composition of other T cell subsets [119]. Early Treg depletion resulted in an increased number of Th17 cells (pro-inflammatory), whereas late depletion led to an increase in Th2 cells, which are associated with promoting fibrosis. These findings suggest that the role of Tregs in IPF may be closely related to both the disease course and disease phenotype. The timing of Treg involvement, as well as the specific subpopulations of Tregs present, appears to influence whether they act to promote or suppress fibrosis. This dual potential of Tregs underscores the need for further investigation to better understand their precise role in IPF and to identify potential therapeutic targets.

### 5.2. B Cells and Autoantibodies

The presence of autoimmunity in idiopathic interstitial pneumonia (IIP), including IPF, has long been recognized. In an effort to create consensus and foster interdisciplinary collaboration, the American Thoracic Society/European Respiratory Society (ATS/ERS) task force published a research statement in 2015 that introduced the concept of “interstitial pneumonia with autoimmune features” (IPAF) [120]. This nomenclature, along with a set of classification criteria, was developed to encompass patients with IIP who exhibit features of autoimmunity but do not meet the diagnostic criteria for a recognized connective tissue disease (CTD). To be classified as IPAF, patients must have one feature from at least two of the three domains: clinical, serologic, and morphologic. The serologic domain includes autoantibodies that are strongly associated with CTDs, such as high-titer ANA (anti-nuclear antibodies), RF (rheumatoid factor), anti-CCP (anti-citrullinated protein antibody), anti-dsDNA (double strand DNA), anti-SSA/SSB (Ro/La), anti-RNP (ribonucleoprotein), anti-Smith, and anti-topoisomerase. Since the introduction of IPAF, several retrospective studies have explored its clinical implications. Small cohort studies found that patients with UIP-IPAF had a similar survival rate to those with IPF [121–123]. A larger prospective study enrolled 376 patients diagnosed with IIP from 28 hospitals in Japan and followed them over a median period of 35 months [124]. Of these patients, 70 (18.6%) met the criteria for IPAF, though only 6 were diagnosed with IPF. This study found that IPAF did not significantly influence the prognosis of IPF patients. Additionally, a post-hoc analysis of the phase III ASCEND trial demonstrated that IPF patients with ANA, RF, and/or anti-CCP antibodies did not exhibit differences in their disease course compared to IPF patients without these autoantibodies [125]. However, due to the relatively small number of IPF patients with IPAF, it remains unclear whether IPF patients who meet the IPAF criteria have a distinct prognosis compared to other IPF patients.

Some small retrospective cohort studies have linked certain autoantibodies with prognostic implications. For example, anti-HSP70 has been associated with near-term lung function deterioration and increased mortality [126], while anti-periplakin, an intermediate filament protein and desmosome component, has been associated with more severe disease but not mortality [127]. In a proteome-wide discovery study, Leuschner et al. identified a high prevalence of autoantibodies in IPF patients through immunoprecipitation of human lung proteome extracts [128]. Despite this, there was broad heterogeneity, with most autoreactivities found in only 1–2 patients, and less than 10% of antigens enriched in five or more patients. Notably, autoantibodies against thrombospondin-1 (THBS1), although identified in only 6 patients (8% of the cohort), were predictive of worse transplant-free survival. However, association does not prove causation, and clinical approaches targeting B cells or autoantibodies in IPF have yet to be validated.

Numerous B-cell markers and soluble factors that promote B-cell proliferation, differentiation, and survival, such as B-cell activating factor (BAFF), have been found to be significantly upregulated in the blood and lungs of patients with IPF [129]. *In vitro* studies have demonstrated that B cells from IPF patients, when stimulated with microbial antigens, can induce fibroblast activation and migration, processes that are also influenced by antifibrotic treatments [130]. However, *in vivo* models have produced conflicting evidence regarding the role of B cells in pulmonary fibrosis. Mice genetically deficient in CD19, a marker that enhances B-cell activation, as well as those with genetic deletion or antibody-mediated depletion of BAFF, have demonstrated protection against bleomycin-induced fibrosis [131,132]. In contrast, μMT knockout mice, which lack mature B cells, did not show protection from fibrosis induced by bleomycin or transforming growth factor-β (TGF-β) [133]. Further, a study by Prele et al. showed that depleting mature B cells using an anti-CD20 antibody (modeled after rituximab) failed to inhibit bleomycin-induced pulmonary fibrosis, despite significantly reducing circulating and lung-resident CD19+ B cells. Instead, the administration of bortezomib significantly reduced fibrosis, which was associated with a notable depletion of CD19 + CD138+ plasma cells in fibrotic areas [134]. These findings suggest that different therapeutic approaches may target varying B cell subsets, which could yield different results depending on the immune cell composition within the fibrotic niche.

In a different clinical setting, triple methods aimed at removing autoantibodies—through the use of therapeutic plasmapheresis, intravenous immunoglobulins (IVIG), and rituximab—have been studied in patients with acute exacerbations of IPF (AE-IPF). These approaches have shown potential clinical benefits, including improved survival and respiratory function in AE-IPF patients [135]. Interestingly, high titers of HEp-2 autoantibodies were associated with better outcomes in these patients. In response to these findings, a randomized trial known as STRIVE-IPF has been designed to further investigate the efficacy of this treatment strategy in AE-IPF [136].

**Figure 2.**
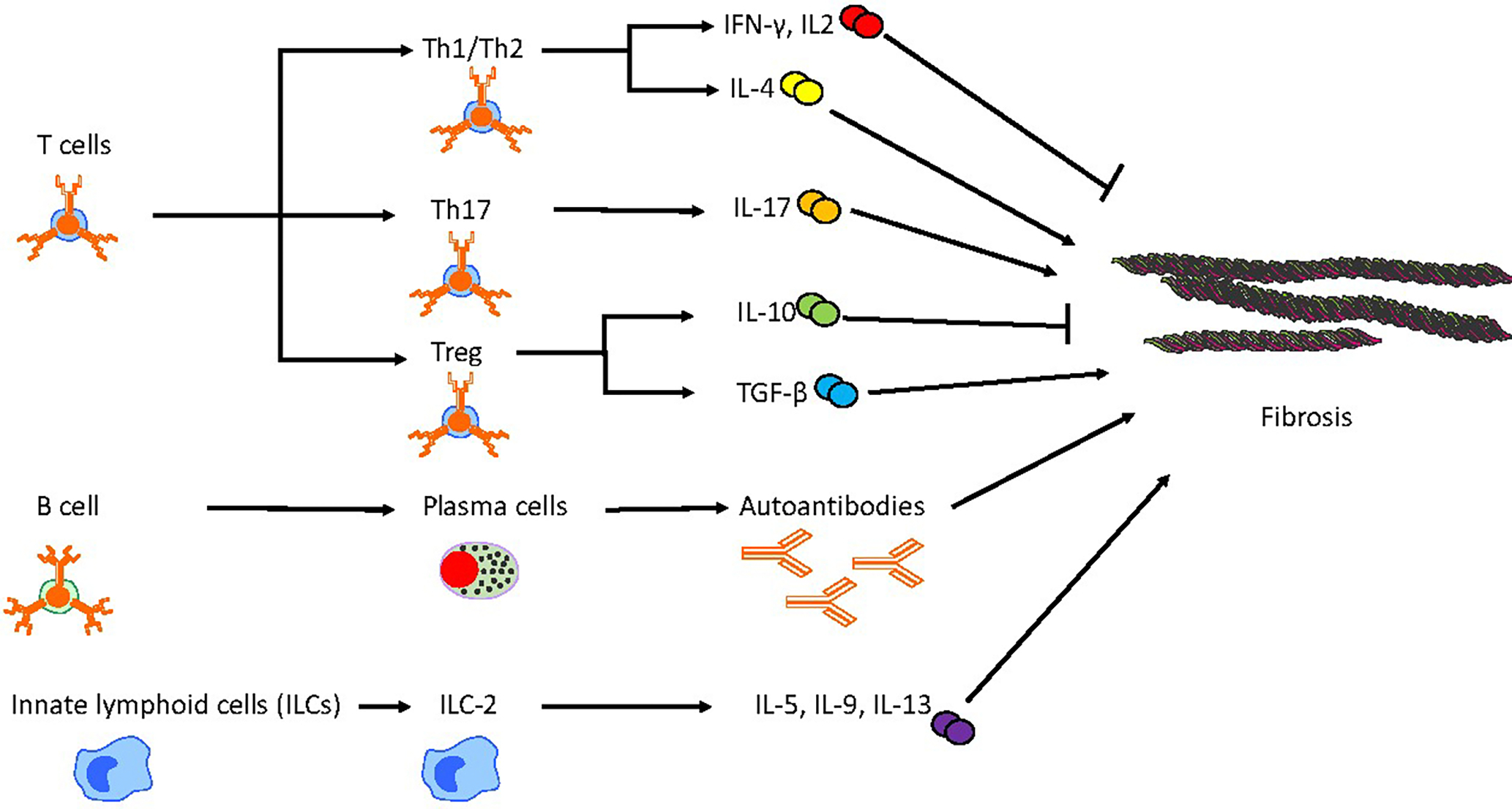
Other adaptive and innate immune cell contributions to pulmonary fibrosis in murine models. This figure depicts the intricate and distinct roles of adaptive and innate immune cell populations in the progression of fibrosis. T helper cells exhibit dual functionality, either promoting or mitigating fibrosis, through differentiation into various subsets and the secretion of distinct cytokines and mediators. B cells contribute to fibrotic processes by differentiating into plasma cells, which produce autoantibodies. Additionally, group 2 innate lymphoid cells (ILC-2) enhance fibrotic progression by secreting profibrotic cytokines.

Figure 2 provides a summary of the adaptive and innate immune cells other than monocytes/macrophages involved in the progression of pulmonary fibrosis in murine models.

## 6. Differences between Human IPF and Animal Models

One of the persistent challenges in pulmonary fibrosis research is the lack of clinical efficacy observed in various treatments that showed promising results in preclinical studies. A potential explanation for this discrepancy lies in the fundamental differences between human idiopathic pulmonary fibrosis and the animal models commonly used to study it.

The discovery that bleomycin, a chemotherapeutic agent, could induce pulmonary fibrosis in humans led to its adoption in experimental models of lung fibrosis, where it has been the most widely used model for over four decades [137]. A single tracheal instillation of bleomycin initiates a well-defined sequence of events and the timeline of these events is clearly established, with epithelial cell death in the early days, inflammation peaking between days 3 and 9, and the fibrotic response reaching its peak around day 14, stabilizing by day 21, and resolving over time [138]. By day 21, signs of tissue repair and regeneration are evident, with Krt8+ alveolar differentiation intermediate (ADI) cells contributing to epithelial regeneration. In contrast, human IPF lungs show arrested alveolar regeneration, with abnormal basaloid (AbBa) cells accumulating in fibrotic regions, failing to differentiate into fully functional alveolar epithelial cells [139]. Thus, due to their acute nature and the pronounced early inflammatory response, animal models fail to faithfully replicate the hallmark features of histologic usual interstitial pneumonia, which is characterized by patchy, dense fibrosis and temporal heterogeneity. In addition, IPF is a disease predominantly affecting the elderly, yet most animal studies are conducted on mice that are 6–8 weeks old, which is approximately equivalent to a 20-year-old human [140,141]. The age-related changes in immune response, tissue repair mechanisms, and susceptibility to fibrosis are not accurately modeled in young animals, which may contribute to the discrepancy between preclinical and clinical results.

Although the bleomycin-induced model has significantly advanced our understanding of the underlying mechanisms of lung fibrosis, key limitations impede its direct translation to human IPF research. It underscores the need for improved animal models that more accurately reflect the chronic, progressive nature of IPF, as well as for more nuanced interpretation of preclinical results.

## 7. Post COVID Pulmonary Fibrosis and IPF

Over the past several years, the world has faced a global pandemic, and only recently have we begun to fully understand the post-acute sequelae associated with COVID-19. While post-COVID pulmonary fibrosis (PCPF) and idiopathic pulmonary fibrosis are distinct diseases with differing etiologies and clinical courses, they share important immunological features that provide insights into overlapping fibrotic mechanisms. For example, histological examination of lungs from patients undergoing transplantation for PCPF revealed extensive immune cell infiltration, predominantly involving CD8+ T cells and macrophages. This was accompanied by significant collagen deposition, a marked reduction in alveolar epithelial cells (AT1 and AT2), and an accumulation of dysplastic epithelial progenitors, suggesting impaired epithelial repair processes [142]. Similarly, analysis of BAL fluid from patients with post-acute sequelae of COVID-19 who exhibited persistent respiratory symptoms and radiographic abnormalities revealed an expansion of monocyte-derived alveolar macrophages. These macrophages displayed a characteristic profibrotic transcriptional program, with upregulated genes such as SPP1 and SPHK1. These findings underscore shared molecular signatures between PCPF and IPF, reinforcing the idea of converging pathogenic pathways across these conditions [143].

Further insights have been gained from experimental models. In an aged mouse model of post-viral pulmonary fibrosis, Narasimhan et al. identified an aberrant immune-epithelial progenitor niche characterized by pathological interactions between immune and epithelial cells. Within this niche, CD8+ T cells secreted IFNγ and TNF, which subsequently activated macrophages to produce IL-1β. This cascade of inflammatory signaling inhibited the differentiation of AT2 cells into AT1 cells, thereby maintaining KRT8^high^ transitional epithelial cells in a dysplastic state. Therapeutically, blocking IFNγ and TNF or neutralizing IL-1β significantly promoted alveolar regeneration and reduced fibrosis, highlighting potential targets for therapeutic intervention [142].

Understanding the transition from acute lung injury to chronic fibrosis in post-COVID cases, particularly the factors distinguishing individuals who develop fibrosis from those who do not, as well as those whose fibrosis resolves versus persists, offers a critical opportunity for IPF research. These insights may reveal novel mechanisms and therapeutic targets, ultimately advancing our understanding of fibrotic diseases and improving patient outcomes.

## 8. Ongoing Trials Targeting Immune Systems in IPF

As understanding of the immune system’s role in idiopathic pulmonary fibrosis (IPF) advances, several clinical trials are investigating immune-targeted therapies to address the disease’s complex pathophysiology. The following is a list of ongoing Phase II and III trials of interventional drugs currently recruiting IPF patients (Table 1). This list excludes information on pre-clinical drugs and drugs studied exclusively outside the United States.

## 9. Perspectives

The immune system’s role in IPF is complex and multidimensional and can shift depending on the stage of the disease. Future research in IPF should focus on understanding the mechanisms that drive immune cell differentiation into either profibrotic or reparative phenotypes within the lung microenvironment by identifying the molecular cues, such as cytokines, chemokines, and cell-surface receptors. Advanced single-cell and spatial transcriptomics and proteomics could elucidate how various signaling pathways influence immune cell differentiation in IPF. Understanding these mechanisms may reveal new ways to reprogram harmful immune cells or promote the expansion of beneficial cell subsets. Key questions remain regarding how immune cells interact with resident lung cells, such as fibroblasts, endothelial cells and epithelial cells, as well as how the inter-regulations among different types of immune cells drive fibrotic remodeling. Additionally, investigating the roles of newly discovered immune subsets, such as ILC2s, could provide insights into how immune cells contribute to fibrosis across various stages and anatomical regions of the lung. The failure of broad immunosuppressive treatments in IPF highlights the need for more precise targeting strategies. A future direction is to design therapies that selectively modulate specific immune cell subsets or signaling pathways without targeting the entire immune response.

Another key area for future research is the timing of immune interventions, as immune cells can play both detrimental and reparative roles depending on the disease stage. Early intervention may help prevent or mitigate fibrosis by targeting inflammatory immune subsets before extensive tissue remodeling begins, while late-stage interventions could focus on modulating rather than suppressing immune activity to preserve reparative functions. Stage-specific biomarkers could help guide the timing of treatments, ensuring interventions are applied when they are most beneficial. Notably, the precise timing of immune intervention is further complicated by the spatial heterogeneity of IPF progression, as the immune system appears to play distinct roles in regions at different stages of the disease. Consequently, an optimal immune-targeting therapeutic strategy for IPF is likely not a one-size-fits-all approach but rather a precise modulation of the immune network, tailored to spatial, temporal, and individual factors.

In conclusion, the intricate interplay between the immune system and idiopathic pulmonary fibrosis underscores the multifaceted nature of this debilitating disease. The immune system’s dual roles—as both a potential contributor to fibrosis and a target for innovative therapies—highlight the complexity of therapeutic interventions. While significant strides have been made in understanding the immunological underpinnings of IPF, many questions remain regarding the specific pathways and cellular interactions driving disease progression. Future research should prioritize a comprehensive approach that integrates advanced molecular techniques, innovative therapeutic strategies, and stage-specific interventions to better address the heterogeneity of IPF. Such efforts could pave the way for precision medicine tailored to individual patient profiles, ultimately improving outcomes and quality of life for those affected by this challenging condition.

## Figures and Tables

**Table 1.** Ongoing Phase II and III Clinical Trials of Interventional Drugs Actively Recruiting Patients with Idiopathic Pulmonary Fibrosis.

Study Medication	Phase of the Clinical Trial	Presumed Mechanism of Action	Primary Outcome	Trial Identifier
BI 1015550	Phase III	selective phosphodiesterase 4B (PDE4B) inhibitor	Absolute change from baseline in Forced Vital Capacity at Week 52	NCT05321069
NAC	Phase III	Anti-inflammatory	Time to one of the following composite endpoint criteria: 10% relative decline in forced vital capacity, first respiratory hospitalization, lung transplant or death from any cause.	NCT04300920
Methyl Prednisone Prednisone	Phase III	Multiple acting targets including innate and adaptive immune system	30-day all-cause mortality in patients with AE-IPF	NCT05674994
TTI 101	Phase II	STAT3 (Signal Transducer and Activator of Transcription 3) inhibitor	Number of Participants with an Adverse Event (AE)	NCT05671835
Axatilimab	Phase II	Monoclonal antibody against colony-stimulating factor-1 receptor (CSF1R)	Annualized rate of decline in morning pre-dose trough forced vital capacity	NCT06132256
Ifetroban	Phase II	Selective thromboxane A2/prostaglandin H2 (TP) receptor antagonist	Change from baseline in Forced Vital Capacity in 12 months	NCT05571059
GRI-0621	Phase II	Dual agonist for the retinoic acid receptors beta (RAR-β) and gamma (RAR-γ)	Safety and Tolerability of oral GRI-0621	NCT06331624
Leramistat	Phase II	Mitochondrial complex I (NADH dehydrogenase) inhibitor	12-week change in Forced vital capacity (FVC)	NCT05951296
LTP001	Phase II	Signal Transducer and Activator of Transcription 3 (STAT3) inhibitor	26-week change from baseline to end of treatment epoch in Forced Vital Capacity expressed in percent predicted	NCT05497284

## Data Availability

Not applicable.
